# Non-invasive prenatal testing of fetal chromosomal aneuploidies: validation and clinical performance of the veracity test

**DOI:** 10.1186/s13039-019-0446-0

**Published:** 2019-07-15

**Authors:** Elena Kypri, Marios Ioannides, Evi Touvana, Ioanna Neophytou, Petros Mina, Voula Velissariou, Spiros Vittas, Alfredo Santana, Filippos Alexidis, Kyriakos Tsangaras, Achilleas Achilleos, Philippos Patsalis, George Koumbaris

**Affiliations:** 1 NIPD Genetics Public Company Ltd, Neas Engomis 31, Engomi, 2409 Nicosia, Cyprus; 2 DNA Diagnosis N Analysis, Adrianoupoleos 1, 55133 Kalamaria, Thessaloniki, Greece; 3 Clinical Genetics Unit, Childhood Hospital Materno-Infantil, Childhood Hospital Materno-Infantil, Las Palmas, GC, Canary Islands Spain; 4 Eurogenetica, Adrianoupoleos 7, 55133 Kalamaria, Thessaloniki, Greece

**Keywords:** Cell-free DNA, Non-invasive prenatal testing, Aneuploidy

## Abstract

**Introduction:**

Non-Invasive Prenatal Testing (NIPT) for fetal aneuploidies using cell-free DNA (cfDNA) has been widely adopted in clinical practice due to its improved accuracy. A number of NIPT tests have been developed and validated. The purpose of this study is to evaluate the performance of the Veracity NIPT test for sex chromosome aneuploidy (SCA) detection in singleton pregnancies, autosomal aneuploidy detection in twin pregnancies and evaluation of Veracity clinical performance under routine NIPT conditions in a diverse cohort.

**Methods:**

Blinded retrospective study in singleton pregnancies (*n* = 305); blinded retrospective and prospective study in twin pregnancies (*n* = 306) and prospective evaluation of clinical performance in singleton and twin pregnancies (*n* = 10564).

**Results:**

Validation study results for the detection of SCAs in singleton pregnancies exhibited 100% sensitivity and specificity and correctly classified 7 (45,X), 4 (47,XXY), 2 (47,XXX) and 1 (47,XYY) cases. Validation study results for autosomal aneuploidy detection in twin pregnancies exhibited 100% sensitivity and specificity and correctly classified 3 trisomy 21, 1 trisomy 18 and 1 trisomy 13 samples. Clinical performance evaluation of Veracity was performed in 10564 pregnancies with median gestational age of 13 weeks, median maternal age 35 years and median gestational weight of 64 kg. Based on confirmation feedback the PPV for trisomies 21, 18 and 13 was estimated at 100% (95% CI, 92–100%), 100% (95% CI, 69–100%) and 71% (95% CI, 29–96%), respectively. Estimated PPV for Monosomy X was 57% (95%CI, 18–90%), while the NPV for SCA detection was estimated at 100% (95% CI, 99.94–100%).

**Conclusion:**

Veracity NIPT test is based on a very powerful, highly accurate methodology that can be safely applied in the clinical setting.

**Electronic supplementary material:**

The online version of this article (10.1186/s13039-019-0446-0) contains supplementary material, which is available to authorized users.

## Introduction

Non-Invasive Prenatal Testing (NIPT) for fetal aneuploidies using cell-free DNA (cfDNA) has been widely adopted in clinical practice due to its improved accuracy, compared to traditional screening approaches using measurements of nuchal translucency and biochemical analytes. Consequently, international bodies including the American Congress of Obstetricians and Gynaecologists (ACOG) and the American College of Medical Genetics and Genomics (ACMG) endorse NIPT as a routine screening option [1, 2].

A number of NIPT tests based on targeted and whole genome-based technologies, mainly employing Next-Generation Sequencing (NGS), have been developed and validated [3–6]. These technologies rely on the ability to detect increases in cfDNA arising from the presence of an extra fetal chromosome. The proportion of cell-free fetal DNA (cffDNA) in maternal circulation (fetal fraction (ff)) is a key determinant of assay performance.

More recently, NIPT has expanded to include the detection of sex chromosome abnormalities (SCAs), and has the potential to offer significant added value to parents and physicians in the form of early detection, outcome preparation and timely treatment. NIPT is also of clinical importance in multiple gestations which pose considerably more difficult management issues, especially in relation to the risks of invasive procedures. Thus, recent studies have focused on the implementation of cfDNA analysis in this pregnancy group. The dizygotic twin group poses additional challenges, because each fetus contributes different amounts of cfDNA in the maternal circulation [7, 8]. Therefore, it is imperative to develop and implement high fidelity NIPT tests which allow accurate fetal fraction quantification and as such can be offered to a wide range of pregnancies.

This study summarizes the Veracity test validation results for the detection of sex chromosome aneuploidies (SCAs) in singleton pregnancies (*n* = 305), the detection of trisomies 13, 18, 21 in twin pregnancies (*n* = 306) and the evaluation of clinical performance for trisomies 13, 18, 21 and X,Y under routine testing conditions in a diverse test cohort (*n* = 10564). Collectively, this report conveys a complete clinical picture of the Veracity NIPT test performance and assesses the implementation and clinical impact on a broader scale.

## Results

For the detection of sex chromosome aneuploidies (SCAs), the clinical performance of Veracity was assessed in a blinded retrospective validation study of a total of 305 plasma samples. The final analysis included samples that fulfilled the fetal fraction threshold of 4% (*n* = 300). In total we detected, 286 normal, 7 Turner (45,X), 4 Klinefelter (47,XXY), 2 Triple X (47,XXX) and 1 47,XYY cases. All cases underwent confirmation by amniocentesis. In the male conceptuses, the test correctly detected 4 /4 47,XXY cases and 1/1 47,XYY case, exhibiting 100% sensitivity and specificity. In the female conceptuses the test correctly detected 7/7 45,X cases and 2/2 47,XXX cases, exhibiting 100% sensitivity and specificity (Table 1).

**Table 1:** Blind validation study results for SCA detection

Karyotype	Number of Samples	Correct call
Normal	286	286 (95% CI, 98.7–100%)
Turner (45,X)	7	7
Klinefelter (47,XXY)	4	4
Triple X (47,XXX)	2	2
47,XYY	1	1

In twin pregnancies, the clinical performance of Veracity was assessed in a blinded validation study (retrospective and prospective) designed to detect fetal trisomies 13, 18 and 21, in a cohort of 306 pregnancies of at least 10 weeks of gestation. Six samples exhibited an insufficient fetal fraction. Trisomy 21 was detected in 3/3 cases, trisomy 18 in 1/1 case and trisomy 13 was detected in 1/1 case (Table 2).

**Table 2:** Blind validation study results in twin pregnancies for trisomy detection

Karyotype	Number of Samples	Correct call
Normal	295	295 (95% CI, 98.8–100%)
Trisomy 21	3	3
Trisomy 18	1	1
Trisomy 13	1	1

**Fig. 1:**
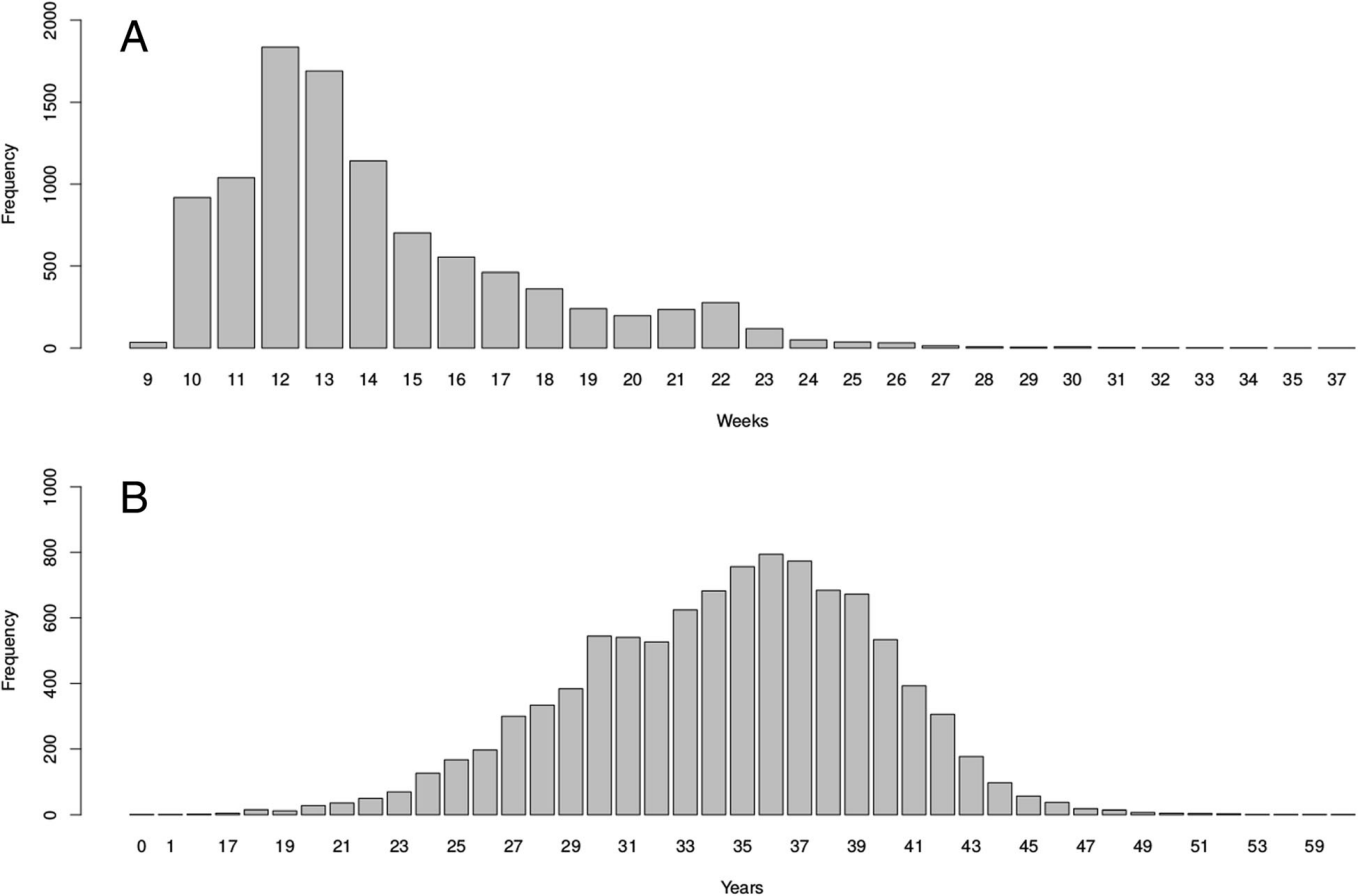
Gestational Age (**a**) and Maternal Age (**b**) Distribution at NIPT sampling

Furthermore, the overall clinical performance of Veracity for the detection of trisomies 13, 18, 21 and SCAs during routine NIPT testing conditions was assessed in a cohort of 10564 mixed-risk samples accessioned by our laboratories until February 2018. The median gestational age in this testing cohort was 13 weeks (IQR4) (Fig. 1a). The distribution of gestational age at the time of sampling indicates that most tested samples were in the first trimester (9–12 weeks) and the beginning of the second trimester (Fig. 1a). The median maternal age was 35 years (IQR7) (Additional file 3: Table S1, Fig. 1b). In the testing population 46.8% of samples were under the age of 35. The median gestational weight was 64 kg (IQR 17) (Additional file 3: Table S1, Additional file 1: Figure S1). The median fetal fraction of reported samples was 9.6% (IQR 5.1) (Additional file 2: Figure S2). In this cohort twin pregnancy samples represented 3% of all referrals.

Out of the 10564 samples, 59.7% were tested for aneuploidies of X and Y, as well as trisomies 13, 18, 21 (Additional file 3: Table S2). The median turn-around time (TAT) for reporting was 5 business days (Additional file 3: Table S2). Insufficient fetal fraction, below a pre-specified threshold of 4%, was reported in 3.7% of the samples. In the singleton cohort the re-draw rate was 3.5%. A repeat sample was received for 71.5% of the low-ff samples and a result was generated for 97.5% of these (Additional file 3: Table S2). The percentage of samples that failed to receive a result after resampling was estimated at 0.07%.

The sample cohort of reported results was 10446. In this cohort, the overall frequency for reported aneuploidies was 1.59% for trisomies 13, 18,21 collectively (Table 3), while the frequency of reported sex chromosome aneuploidies was 0.58% (Table 3). To assess accuracy, follow-up was requested from referring centres (based on confirmatory testing using amniocentesis for abnormal samples and live birth examination for normal samples). Follow-up confirmation results were available for 44 samples of trisomy 21, 10 trisomy 18, 7 trisomy 13. Based on the confirmation feedback the PPV for trisomies 21, 18, 13 was estimated at 100% (95% CI, 92–100%), 100% (95% CI, 69–100%) and 71% (95% CI, 29–96%) respectively (Table 3). Follow-up results for sex chromosome aneuploidies was limited. Estimated PPV for Monosomy X was estimated at 57% (95% CI, 18–90%) while the NPV for SCA detection was estimated at 100% (95% CI, 99.94–100%) (Table 3).

**Table 3:** Veracity clinical Performance

NIPT result for aneuploidies (T21, T18, T13)	Number	Follow-up	Correct	Specificity	NPV
Normal	10280	10280	10280	99.98% (95% CI, 99.93–99.998%)	100% (95% CI, 99.96–100%)
NIPT result for aneuploidies (T21, T18, T13)	Number	Follow-up	Correct	Sensitivity	PPV
Trisomy 21	126	44	44	100% (95% CI, 92–100%)	100% (95% CI, 92–100%)
Trisomy 18	24	10	10	100% (95% CI, 69–100%)	100% (95% CI, 69–100%)
Trisomy 13	16	7	5	100% (95% CI, 48–100%)	71% (95% CI, 29–96%)
NIPT result for SCAs	Number	Follow-up	Correct	Specificity	NPV
Normal	6200	6200	6200	99.95% (95% CI, 99.86–99.99%)	100% (95% CI, 99.94–100%)
NIPT result for SCAs	Number	Follow-up	Correct	Sensitivity	PPV
Monosomy Χ	16	7	4	100% (95% CI, 40–100%)	57% (95% CI, 18–90%)
Trisomy X	6	2	2	–	–
47, ΧΧΥ	10	4	4	–	–
47, ΧΥΥ	3	0	–	–	–
48, ΧXYY	1	1	1	–	–

## Discussion

A major objective in the field of prenatal testing is the reduction of the number of unnecessary invasive procedures and this is mainly achieved with the use of NIPT. Veracity is a targeted NIPT test that enables targeted analysis of regions of clear clinical relevance in combination with a highly accurate fetal fraction estimation algorithm [5]. In addition to common autosomal trisomies, Veracity can perform NIPT for sex chromosome aneuploidies which include Turner (45,X), Klinefelter (47,XXY), Triple X (47,XXX) and 47,XYY and 48,XXYY constitutions. Although individually each condition is relatively rare, cumulatively SCAs occur in approximately 0.3% of all live births [9]. In this validation study which included 300 reported samples, the assay exhibited 100% sensitivity and specificity and correctly classified 7 Turner (45,X), 4 Klinefelter (47,XXY), 2 Triple X (47,XXX) and 1 47,XYY cases. The data from this cohort of SCAs demonstrates robust testing performance in SCAs and similar to autosomal aneuploidies [5].

The test’s performance was also assessed for common autosomal aneuploidy detection in twin pregnancies, employing a cohort of 306 twin pregnancies. In this study which included 300 reported samples, the assay exhibited 100% sensitivity and specificity and correctly classified all abnormal samples.

Furthermore, the data from the large, multi-center prospective study illustrate the clinical performance of Veracity under routine NIPT conditions, in a large pregnancy cohort in a diverse, mixed-risk population, including singleton and twin pregnancies, as well as pregnancies achieved with in-vitro fertilization (IVF), vanished twin and oocyte donation and demonstrated high sensitivity and specificity. Collectively, data from this work is consistent with previous validation results [5] and show Veracity to be a powerful, highly accurate, cost effective, automated high performance NIPT assay, that can be applied in a wide range of pregnancies in the clinical setting.

The targeted assay described here is inherently characterized by high depth of sequencing, which allows highly accurate fetal fraction quantification and aneuploidy detection. In the clinical setting accurate fetal fraction quantification is of paramount importance for accurate classification [10, 11]. Accurate fetal fraction estimation is also crucial in twin pregnancy testing. In this study, we applied an algorithm for fetal fraction estimation in dichorionic twins, which measures the lower fetal fraction of the two fetuses, rather than the total fetal fraction. This approach was specifically designed to alleviate potential limitations of other methods which necessitate strong assumptions regarding the cfDNA contributions of the two fetuses that may not apply to all clinical samples. As such, we ensure that the lowest possible fetal fraction of each sample is considered for classification purposes, thus minimizing the possibility of incorrect classification that could arise from low proportions of fetal DNA by one of the two fetuses.

In contrast to many previous studies, the cohort of samples in the prospective study of 10564 samples was diverse and included twin pregnancies as well as IVF and egg donor pregnancies. The study included pregnant women of all risk levels with 46.8% under the age of 35. This age distribution indicates the broader applicability of NIPT in the general population.

Approximately 3.7% of samples in the clinical performance study presented a fetal fraction below 4%. We observed that a high proportion of such samples was obtained from participants with higher body weight. The median weight of samples with fetal fraction below the threshold was 78 kg whereas for samples with fetal fraction above threshold was 64 kg (*p* value < 0.00001). This finding is in accordance with previously published data [12, 13]. Although the correlation of BMI with fetal fraction would lead to a more informative conclusion, the height information was not available for all samples in this dataset. Our data indicate that repeat cfDNA testing is effective in providing reliable results after initial failure due to low ff. Veracity repeat cfDNA testing was more effective than previously published meta-analysis studies using alternative methodologies [14]. In this context it is important that the assay estimates ff accurately and that practitioners develop practice guidelines to consider the fetal fraction, maternal weight, gestational age and other clinical pregnancy indications in recommending repeat testing. Furthermore, the Veracity analysis pipeline allows for a clear separation between the risk scores of trisomic and disomic samples allowing a binary classification scheme which prevents unclassified, inconclusive or ‘boarderline’ results reported using other methodologies [15].

The overall performance of Veracity in the sample cohort of this prospective study, indicated 100% (95% CI, 94–100%) sensitivity for all common trisomies. Overall, demonstrated sensitivities indicate a more robust performance of Veracity compared to other methodologies, as published in multiple meta-analysis studies [15, 16] . This could be attributed to the underlying technology platform that ensures extremely high accuracy by enabling robust fetal fraction estimation and by avoiding copy number variants, or other complex genomic architectural elements which can cause false positive (FP) or false negative (FN) results [17].

In the present prospective study, we determined the PPV for trisomy 13 and monosomy X to be 71 and 57% respectively. The lower PPV for trisomy 13 and monosomy X is expected and is concordant with results from previously reported studies from large scale cytogenetic analysis [18, 19]. There are known biological reasons that are responsible for discrepant NIPT results. Most false positive results for trisomy 13 and monosomy X are a consequence of confined placental mosaicism (CPM). As previously reported, CPM has a higher incidence in monosomy X and trisomy 13 [18, 19], which likely explain the lower observed PPV for trisomy 13 and monosomy X. Other reasons for such discrepancies may include an undetected vanishing twin, maternal chromosome abnormality (mosaic or otherwise) and maternal metastatic disease. In the present prospective study, follow-up information of normal samples was based only on live birth examination. This poses a limitation, as the calculated NPV value could be lower due to the presence of undetected SCA constitutions.

## Conclusion

The application of NIPT has revolutionized prenatal screening of common aneuploidies and other conditions of clinical relevance. This study provides further proof of the high accuracy of NIPT compared to conventional screening methods. The role of first trimester nuchal translucency measurement and conventional biochemical testing needs to be reassessed in the context of the use of cfDNA testing which is a powerful tool in prenatal care. Furthermore, we hereby show that Veracity, a new NIPT test based on a novel technology which was developed to overcome many of the limitations of other NIPT tests, exhibits high accuracy both in validation studies and routine testing conditions. The test’s high read depth and ability to efficiently capture cell- free DNA fragments delivers state-of the-art performance in fetal aneuploidy detection, fetal fraction estimation and cost effectiveness.

With the advent and broad implementation of these powerful NIPT technologies, challenges related to pretest counseling issues, objective assessment of performance, the interpretation of results and counseling-related issues do arise and need to be addressed. Conclusively, NIPT is a powerful tool that provides clinicians and prospective parents with important information and empowers them to make informed decisions regarding pregnancy management.

## Methods

Blinded retrospective and prospective studies.

### Subjects

All samples collected retrospectively were obtained anonymously from pregnant women of at least 18 years of age from the 10th week of gestation from multiple referring centres. Protocols used for sample collection were approved by the National Bioethics Committees and informed consent was obtained from all participants. All samples collected prospectively were collected after the 9th week of gestation and sent to our laboratories. Appropriate informed consent was obtained from all subjects included in this study.

A total of 305 singleton pregnancy samples were included in the blinded retrospective validation study for SCAs detection. All samples were confirmed by invasive testing. Five samples exhibited insufficient fetal fraction and were excluded from analysis. The sex chromosome abnormality cases enrolled in this study included 45,X, 47,XXY, 47,XXX, and 47,XYY cases.

A total of 306 twin pregnancy samples were included in the blinded validation study for aneuploidy detection of trisomy 13, 18, 21. This cohort included abnormal cases for these trisomies. A subset of 100 samples was collected in a blinded retrospective manner. All samples were confirmed by invasive testing. A subset of 206 twin samples was collected prospectively. In this cohort, confirmation feedback was available for abnormal samples. Six samples out of the 306 exhibited insufficient fetal fraction and were excluded from analysis.

### Sample collection and preparation

A mean of 8 ml of peripheral blood was collected from each subject into EDTA-containing tubes or cell-free DNA BCT tubes (Streck Inc.; Omaha, NE). A mean of 4 ml of plasma was isolated using a double centrifugation protocol by centrifugation at 1600 *g* for 10 min, followed by 16000 *g* for 10 min. Isolated plasma samples were stored at − 80 °C until subsequent analysis. Samples were coded to all operators and the analysts who processed the samples. Circulating cell-free DNA (cfDNA) was extracted from 4 ml of plasma using the Qiasymphony DSP Virus or Qiasymphony Circulating DNA Kit (Qiagen, Valencia, CA). Samples were processed for library preparation and were enriched using a target capture protocol as previously described using custom target capture sequences (TACS) of approximately 250 bp designed to capture selected loci on chromosome 21, chromosome 18 and chromosome 13, chromosome X and chromosome Y [5]. Captured sequences were eluted and amplified using outer-bound adaptors. Enriched products were pooled and sequenced on the Illumina, Nextseq 500 sequencing platform.

### Data analysis

Median and interquartile range (IQR) was used as descriptive statistics. The IQR is considered as a measure of statistical dispersion, being equal to the difference between the third (upper) quartile and the first (lower) quartile, with the term quartile being used to describe the division of our data set into four equal portions (quartiles). The IQR value provides useful information on the spread of those data points that (i) lie around the median and (ii) account for the 50% of the total number of data points. The larger the IQR value the larger is the spread.

Custom target capture sequences (TACS) were designed to capture selected loci on chromosomes 21, 18, 13, X and Y with approximately 500 TACS per chromosome. Classification of fetal aneuploidy was performed as previously described with modifications [5]. In brief, multiple aneuploidy detection engines and different statistical approaches were used to determine the sample’s risk for aneuploidy. The fetal fraction of each sample was also estimated using methods that are previously described in [5]. In dichorionic twins, the algorithm estimated the fetal fraction of the fetus with the smallest contribution of fetal material towards the total fetal content, by taking into account the marginal contribution of each fetus to the total fetal fraction. A metropolis-Hastings algorithm was implemented to get samples from the marginal posterior probability distributions.

Results were compiled electronically and were reviewed by a subject matter expert who assigned the final classification.

### Veracity analytical performance evaluation

All data included in this prospective study were generated in our CLIA certified and CAP accredited laboratory until February 2018. Testing was performed in whole blood samples that were collected in cell-free DNA BCT tubes (Streck Inc.; Omaha, NE) after the 9th week of gestation and sent to our laboratories by 176 different referring centres from 21 countries. Appropriate informed consent was obtained from all subjects included in this study. Plasma was isolated as described above. Upon reception and sample processing, samples were evaluated for initial laboratory quality metrics. Samples were rejected if the tubes were broken, blood volume was too low, insufficiently labelled or if the sample was clotted, grossly hemolysed or arrived in the laboratory more than 8 days after collection. A redraw was requested for samples that failed to meet initial laboratory quality control criteria. Samples that failed quality control criteria were re-drawn and analysed. A re-draw sample was also requested for samples that failed to meet a sufficient fetal fraction threshold (*n* = 390). Consultation was provided by our laboratory personnel for the collection of a subsequent sample. All results were reviewed by the laboratory director prior to the final reporting of results to the referring physician. Confirmation feedback and follow-up outcome was either requested by a laboratory director or provided voluntarily by the referring centres including details for the confirmation method. The confidence intervals were computed using the Clopper and Pearson method and reflect the additional uncertainty from exclusion of no-follow up cases. The assumption is that the expected value of the true proportions (sensitivity, specificity and PPV) do not change between the total number of positive calls and the subset for which we have follow-up.

## Additional file

Additional file 1: **Figure S1**. Maternal Weight Distribution at NIPT sampling. (TIFF 156 kb)

Additional file 2: **Figure S2**. Distribution of fetal fraction estimates. (TIFF 60 kb)

Additional file 3: **Table S1**. Patient Demographics. **Table S2**. Test Performance. (DOCX 14 kb)

## Data Availability

All supporting data are included in the manuscript.

## Abbreviations

ACMG: American College of Medical Genetics and Genomics
ACOG: American Congress of Obstetricians and Gynaecologists
cfDNA: cell free DNA
cffDNA: cell free fetal DNA
CPM: Confined Placental Mosaicism
FN: False Negative
FP: False Positive
IQR: Interquartile range
IVF: In-vitro fertilization
NGS: Next-Generation Sequencing
NIPT: non invasive prenatal testing
NPV: Negative Predictive Value
PPV: Positive Predictive Value
SCA: Sex chromosome aneuploidies
T13: trisomy 13
T18: trisomy 18
T21: trisomy 21
TACS: Target Capture sequences
TAT: Turn-around time

## References

[1] Gregg Anthony R., Skotko Brian G., Benkendorf Judith L., Monaghan Kristin G., Bajaj Komal, Best Robert G., Klugman Susan, Watson Michael S. (2016). Noninvasive prenatal screening for fetal aneuploidy, 2016 update: a position statement of the American College of Medical Genetics and Genomics. Genetics in Medicine.

[2] ACOG (2015). Cell- free DNA screening for fetal aneuploidy. Obstet Gynecol.

[3] Chiu RWK, Chan KCA, Gao Y, Lau VYM, Zheng W, Leung TY (2008). Noninvasive prenatal diagnosis of fetal chromosomal aneuploidy by massively parallel genomic sequencing of DNA in maternal plasma. Proc Natl Acad Sci U S A [Internet]..

[4] Chitkara U, Hudgins L, Fan HC, Blumenfeld YJ, Chitkara U, Hudgins L, et al. Noninvasive diagnosis of fetal aneuploidy by shotgun sequencing DNA from maternal blood. Proc Natl Acad Sci U S A [Internet] 2008;105(42):16266–16271. Available from: https://www.ncbi.nlm.nih.gov/pmc/articles/PMC2562413/. 10.1073/pnas.0808319105 PMC2562413 18838674

[5] Koumbaris G., Kypri E., Tsangaras K., Achilleos A., Mina P., Neofytou M., Velissariou V., Christopoulou G., Kallikas I., Gonzalez-Linan A., Benusiene E., Latos-Bielenska A., Marek P., Santana A., Nagy N., Szell M., Laudanski P., Papageorgiou E. A., Ioannides M., Patsalis P. C. (2016). Cell-Free DNA Analysis of Targeted Genomic Regions in Maternal Plasma for Non-Invasive Prenatal Testing of Trisomy 21, Trisomy 18, Trisomy 13, and Fetal Sex. Clinical Chemistry.

[6] Stokowski R, Wang E, White K, Batey A, Jacobsson B, Brar H (2015). Clinical performance of non-invasive prenatal testing (NIPT) using targeted cell-free DNA analysis in maternal plasma with microarrays or next generation sequencing (NGS) is consistent across multiple controlled clinical studies. Prenat Diagn.

[7] Struble CA, Syngelaki A, Oliphant A, Song K, Nicolaides KH (2014). Fetal fraction estimate in twin pregnancies using directed cell-free DNA analysis. Fetal Diagn Ther.

[8] Canick JA, Kloza EM, Lambert-Messerlian GM, Haddow JE, Ehrich M, van den Boom D (2012). DNA sequencing of maternal plasma to identify Down syndrome and other trisomies in multiple gestations. Prenat Diagn.

[9] Morris JK, Alberman E, Scott C, Jacobs P (2008). Is the prevalence of Klinefelter syndrome increasing?. Eur J Hum Genet.

[10] Benn P, Cuckle H (2014). Theoretical performance of non-invasive prenatal testing for chromosome imbalances using counting of cell-free DNA fragments in maternal plasma. Prenat Diagn.

[11] Wright D., Wright A., Nicolaides K. H. (2014). A unified approach to risk assessment for fetal aneuploidies. Ultrasound in Obstetrics & Gynecology.

[12] Wang E, Batey A, Struble C, Musci T, Song K, Oliphant A (2013). Gestational age and maternal weight effects on fetal cell-free DNA in maternal plasma. Prenat Diagn.

[13] Ashoor G, Syngelaki A, Wagner M, Birdir C, Nicolaides KH (2012). Chromosome-selective sequencing of maternal plasma cell-free DNA for first-trimester detection of trisomy 21 and trisomy 18. Am J Obstet Gynecol.

[14] Palomaki GE, Kloza EM. Prenatal cell-free DNA screening test failures: a systematic review of failure rates, risks of Down syndrome, and impact of repeat testing. Genet Med [Internet]. 2018; Available from:http://www.nature.com/doifinder/10.1038/gim.2018.22 10.1038/gim.2018.22 30514979

[15] Taylor-Phillips Sian, Freeman Karoline, Geppert Julia, Agbebiyi Adeola, Uthman Olalekan A, Madan Jason, Clarke Angus, Quenby Siobhan, Clarke Aileen (2016). Accuracy of non-invasive prenatal testing using cell-free DNA for detection of Down, Edwards and Patau syndromes: a systematic review and meta-analysis. BMJ Open.

[16] Gil MM, Quezada MS, Revello R, Akolekar R, Nicolaides KH (2015). To C. analysis of cell-free DNA in maternal blood in screening for fetal aneuploidies: updated meta-analysis. Ultrasound Obs Gynecol.

[17] Snyder Matthew W., Simmons LaVone E., Kitzman Jacob O., Coe Bradley P., Henson Jessica M., Daza Riza M., Eichler Evan E., Shendure Jay, Gammill Hilary S. (2015). Copy-Number Variation and False Positive Prenatal Aneuploidy Screening Results. New England Journal of Medicine.

[18] Grati Francesca (2014). Chromosomal Mosaicism in Human Feto-Placental Development: Implications for Prenatal Diagnosis. Journal of Clinical Medicine.

[19] Grati FR, Bajaj K, Zanatta V, Malvestiti F, Malvestiti B, Marcato L (2017). Implications of fetoplacental mosaicism on cell-free DNA testing for sex chromosome aneuploidies. Prenat Diagn.

