# Supplementary figures and images for: Non-invasive prenatal testing of fetal chromosomal aneuploidies: validation and clinical performance of the veracity test
Source: Mol Cytogenet. 2019 Jul 15;12:34. doi: 10.1186/s13039-019-0446-0 (PMC6628499; doi:10.1186/s13039-019-0446-0)

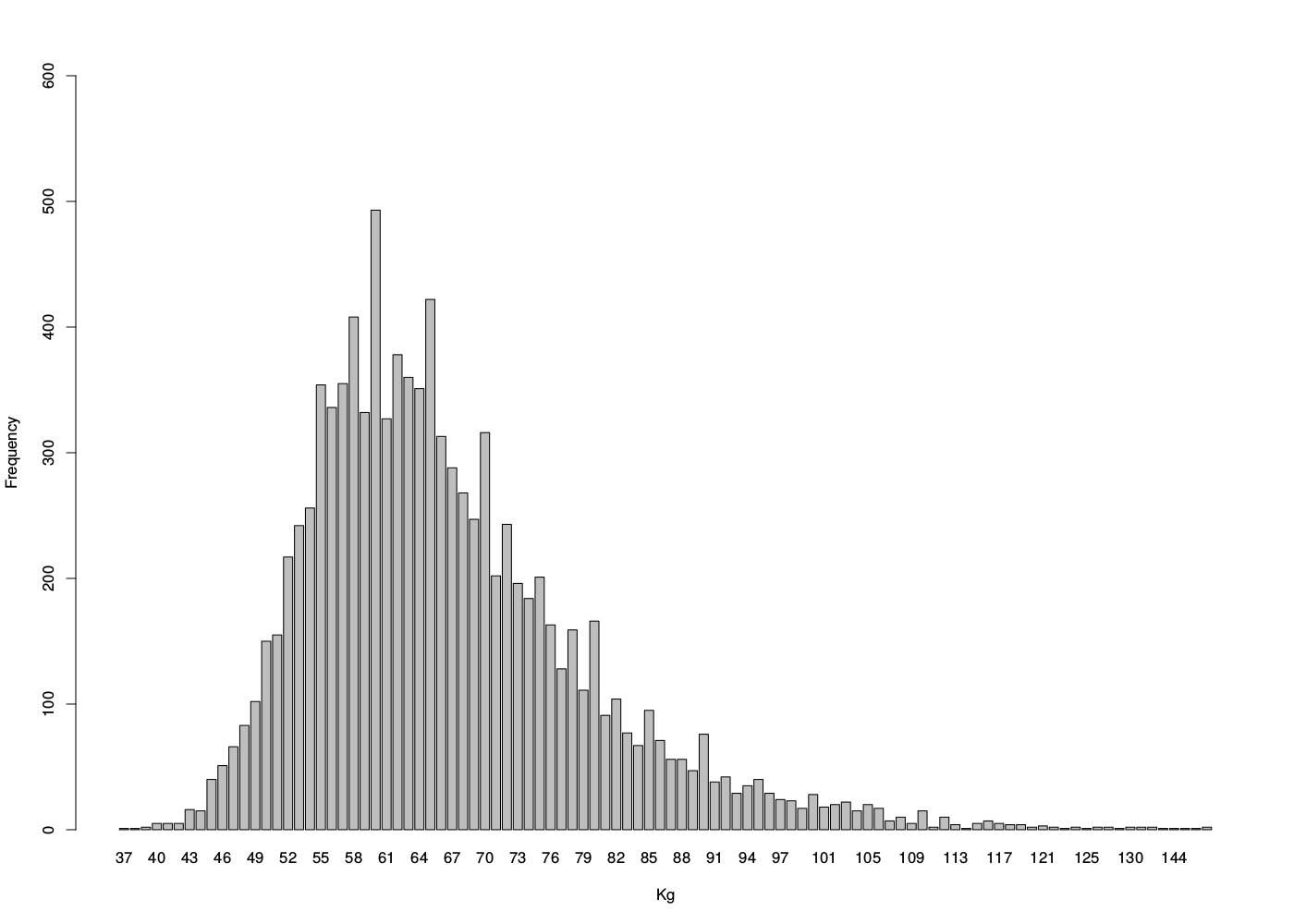

Supplement: Supplementary file 1 — Figure S1. Maternal Weight Distribution at NIPT sampling. (TIFF 156 kb) [file 13039_2019_446_MOESM1_ESM.tiff]

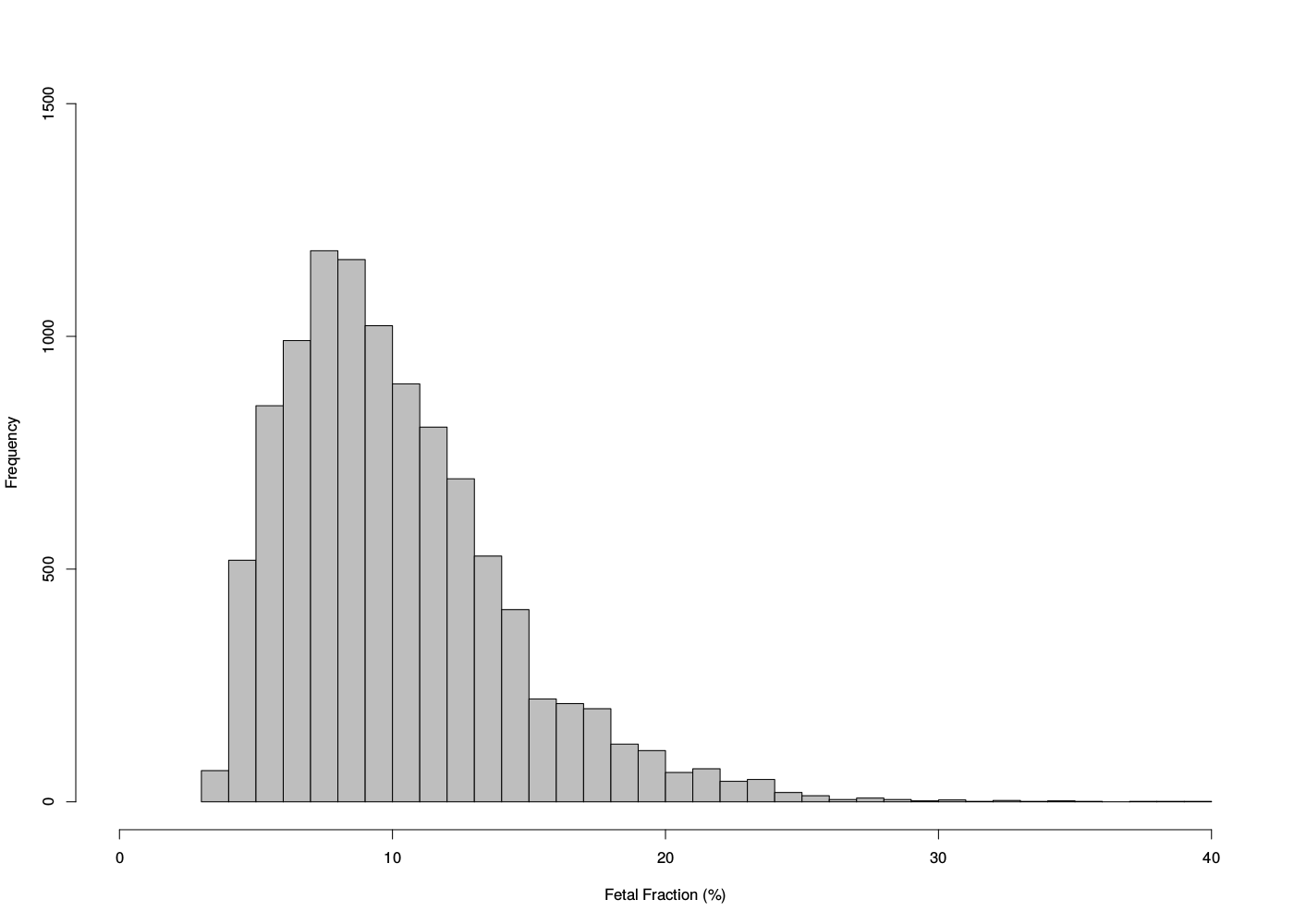

Supplement: Supplementary file 2 — Figure S2. Distribution of fetal fraction estimates. (TIFF 60 kb) [file 13039_2019_446_MOESM2_ESM.tiff]
