# Supplementary material for: Non-invasive prenatal testing of fetal chromosomal aneuploidies: validation and clinical performance of the veracity test
Source: Mol Cytogenet. 2019 Jul 15;12:34. doi: 10.1186/s13039-019-0446-0 (PMC6628499; doi:10.1186/s13039-019-0446-0)
Supplement: Supplementary file 3 — Table S1. Patient Demographics. Table S2. Test Performance. (DOCX 14 kb) [file 13039_2019_446_MOESM3_ESM.docx]

Supplementary Tables

| **Table S1: Patient Demographics** |
| --- |
| Maternal Age 35 years |
| Gestational Age 13 weeks |
| Multifetal Gestations 3% |
| Maternal Weight 64 kg |

| **Table S2: Test Performance** |
| --- |
| Requested Test with SCAs 59.7% |
| Turnaround time 5 business days |
| Low fetal fraction samples 3.7% |
| Redraws received: 71.5% |
| Reported after re-draw 97.5% |
